# Expression and Regulation of the Endogenous Retrovirus 3 in Hodgkin’s Lymphoma Cells

**DOI:** 10.3389/fonc.2013.00179

**Published:** 2013-07-10

**Authors:** Stefanie Kewitz, Martin Sebastian Staege

**Affiliations:** ^1^Department of Pediatrics, Martin-Luther-University Halle-Wittenberg, Halle, Germany

**Keywords:** Hodgkin’s lymphoma, gene expression, endogenous retrovirus ERV3, histone deacetylase inhibitor, hypoxia-mimetic cobalt(II) chloride

## Abstract

Human endogenous retroviruses (ERV) are an integral part of our genome. Expression of ERV is usually switched off but reactivation of ERV has been observed in varying human diseases including cancer. Recently, reactivation of ERV associated promoters in Hodgkin’s lymphoma (HL) cells has been described. Despite relatively good prognosis, not all patients with HL can be cured with the established therapy and this therapy is associated with severe late side effects. Therefore, new targets are required for the development of future treatment strategies. Reactivated ERV might represent such target structures. Therefore, we asked which ERV loci are expressed in HL cells. Using DNA microarray analysis, we found no evidence for a general activation of ERV transcription in HL cells. In contrast, we observed down-regulation of ERV3, an ERV with potential tumor suppressor function, in HL cells in comparison to normal blood cells. Interestingly, ERV3 was also differentially expressed in published DNA microarray data from resting versus cycling B cells. Treatment of HL cells with the histone deacetylase inhibitor vorinostat strongly up-regulated ERV3 expression. In addition, we observed up-regulation in HL cells after treatment with hypoxia-mimetic cobalt(II) chloride. Like vorinostat, cobalt(II) chloride inhibited cell growth of HL cells. Our results suggest that cell cycle inhibition of HL cells is accompanied by up-regulation of ERV3.

## Introduction

The exact etiology of Hodgkin’s lymphoma (HL) is unknown, but immunological and molecular properties suggest that the majority of HL are derived from B cells (1, 2). HL cells have a characteristic gene-expression profile that discriminates these cells from other normal and transformed cells (3, 4). Especially for pediatric HL patients the prognosis is relatively good, and with the combination of radio- and chemo-therapy the majority of patients with HL can be cured. However, the established therapy is associated with a plethora of late adverse side effects and some patients with chemo-resistant disease cannot be cured (5, 6). Therefore, it is important to search for new targets for treatment of patients with HL.

Recently, reactivation of endogenous retrovirus (ERV) activity has been observed in HL (7). This reactivation leads to expression of the receptor for macrophage colony-stimulating factor (colony-stimulating factor 1 receptor, CSF1R), a known oncogene, in HL cells. ERV are an integral part of the genome of virtually all eukaryotes, and ERV loci have been extensively analyzed in plants, insects, and vertebrates (8–13). In the human genome, ERV derived sequences constitute at least 8% of the complete DNA. Usually, ERV are silenced epigenetically and are not transcribed into RNA. Reactivation of ERV has been observed under pathological conditions, e.g., in cancer cells. Such reactivation can result even in the formation of virus particles (14, 15). A small number of ERV-encoded proteins can be found under normal physiological conditions. Such proteins can exert variable biological functions (16, 17). One notable example is ERVW-1 (also known as syncytin 1) which is required for proper formation of the syncytial layer of the placenta (16). In this ERV only the envelope protein is functional. Other open reading frames (ORF) have been inactivated by deleterious mutations. Such mutations destroy the ORF of the majority of ERV. Some ERV with intact ORF encode superantigens (18, 19). Such superantigens can activate a high percentage of all T cells. The activation of these T cells can lead to hyper-reactivity of the immune system but can also lead to the final deletion of the activated T cells.

In addition to the potential involvement in the pathogenesis of human diseases, ERV might also represent interesting target structures for the development of future treatment strategies. Immune responses against ERV-encoded antigens have been described in cancer patients (20, 21). In melanoma patients, antibodies against ERV are associated with shorter disease free survival (21). On the other hand, cytotoxic T cells with specificity for ERV-encoded antigens can kill melanoma cells (22), colorectal cancer cells (23), and renal cancer cells (24). In addition, antibodies against ERV products can inhibit growth of breast cancer cells *in vitro* and in an animal model (25). Successful immunization of rhesus macaques against simian ERV suggests that ERV derived antigens can be used as safe vaccines without development of auto-immunity (26). Interestingly, ERV-specific T cells have been detected in patients after allogeneic hematopoietic stem cell transplantation (alloHSCT) (24). These T cells can kill the tumor cells and might be responsible for graft-versus-tumor effects after alloHSCT (24). Graft-versus-tumor effects have also been described in HL patients after alloHSCT (27). It remains unclear whether ERV reactivation in HL cells (7) has an impact on such graft-versus-tumor effects. We asked whether ERV reactivation in HL is a phenomenon affecting multiple (or all) ERV loci or whether this reactivation is specific for single ERV loci. Therefore, we used DNA microarray data for the analysis of multiple ERV loci in HL cells. DNA microarrays can be used for the characterization of complete gene-expression profiles from normal and malignant cells in a single experiment (28). Modern DNA exon microarrays contain several probe sets with specificity for ERV and, therefore, can be used for analysis of expression of multiple ERV loci at once.

## Materials and Methods

### Cell lines and cell culture

Hodgkin’s lymphoma-cell lines HDLM-2, KM-H2, L-1236, L-428, and L-540 (29–33) were obtained from the Deutsche Sammlung von Mikroorganismen und Zellkulturen (DSMZ), Braunschweig, Germany. P439-6 cells were kindly provided by G. W. Bornkamm and G. Laux, Munich, Germany. P493-6 cells carry an EBV nuclear antigen 2 (EBNA2)-estrogen receptor fusion gene and *MYC* under control of a promoter which can be regulated by tetracycline (34–36). All cells were cultured in RPMI-1640 (Invitrogen, Karlsruhe, Germany) supplemented with 10% fetal calf serum, 100 U/mL penicillin, and 100 μg/mL streptomycin (PAA, Pasching, Germany) at 37°C in a humidified atmosphere at 5% CO_2_. Treatment of HL cells with 1 μM vorinostat was carried out as described (37) at a cell density of 1 × 10^6^ cells/mL for 24 h. Dimethyl sulfoxide (DMSO) was used as control. For simulation of hypoxia, HL cells were treated for 2 days at a cell density of 1 × 10^6^ cells/mL with 200 μM cobalt(II) chloride. P439-6 cells were cultured for 4 days in medium with or without 2 μM estradiol and/or 1 μg/mL tetracycline.

### Gene-expression analysis

RNA from cell lines were isolated using TriFast reagent (peqlab, Erlangen, Germany) following manufacturer’s protocol. Two micrograms of the RNA were transcribed into cDNA and used as template for polymerase chain reaction (PCR). The following primer combinations were used for real-time quantitative reverse transcription-PCR (qRT-PCR): actin beta (ACTB): 5′-GGC ATC GTG ATG GAC TCC G-3′, 5′-GCT GGA AGG TGG ACA GCG A-3′; ERV3: 5′-GGG AGT ATG CGG AAA GTT CA-3′, 5′-CTC CAA GGG ATG AGA ACC AA-3′. Quantitative RT-PCR was performed using the Go Taq qPCR Master Mix (Promega, Mannheim, Germany). The reaction was performed with 10 μL Go Taq qPCR Master Mix, 6 μL water, 1 μL primer combination, and 2 μL cDNA using the following conditions: 94°C, 30 s; 60°C, 30 s; 72°C, 45 s (40 cycles). Determination of gene expression was performed using the $2^{-\Delta \Delta C_{t}}$ method (38). Global gene expression in HL cell lines was analyzed by using Affymetrix Human Exon 1.0ST arrays (Affymetrix, Santa Clara, USA). In addition to microarray data from HL cell lines L-540, HDLM-2 and L-428 (39), microarray data from normal peripheral blood cells (40), P493-6 cells (41), and normal B cells (42, 43) were used for comparative analysis. These cel files were down-loaded from the gene-expression omnibus (GEO) data base. All cel files were processed together using the robust microarray analysis (RMA) algorithm with Expression Console 1.1 (Affymetrix). Cel files from DNA microarrays from HL cell lines have been submitted to the GEO data base (accession number GSE47686). Signal intensities from ERV-specific probe sets were visualized with the Genesis software (44).

## Results

### Analysis of ERV expression in DNA microarray data

**Figure 1:**
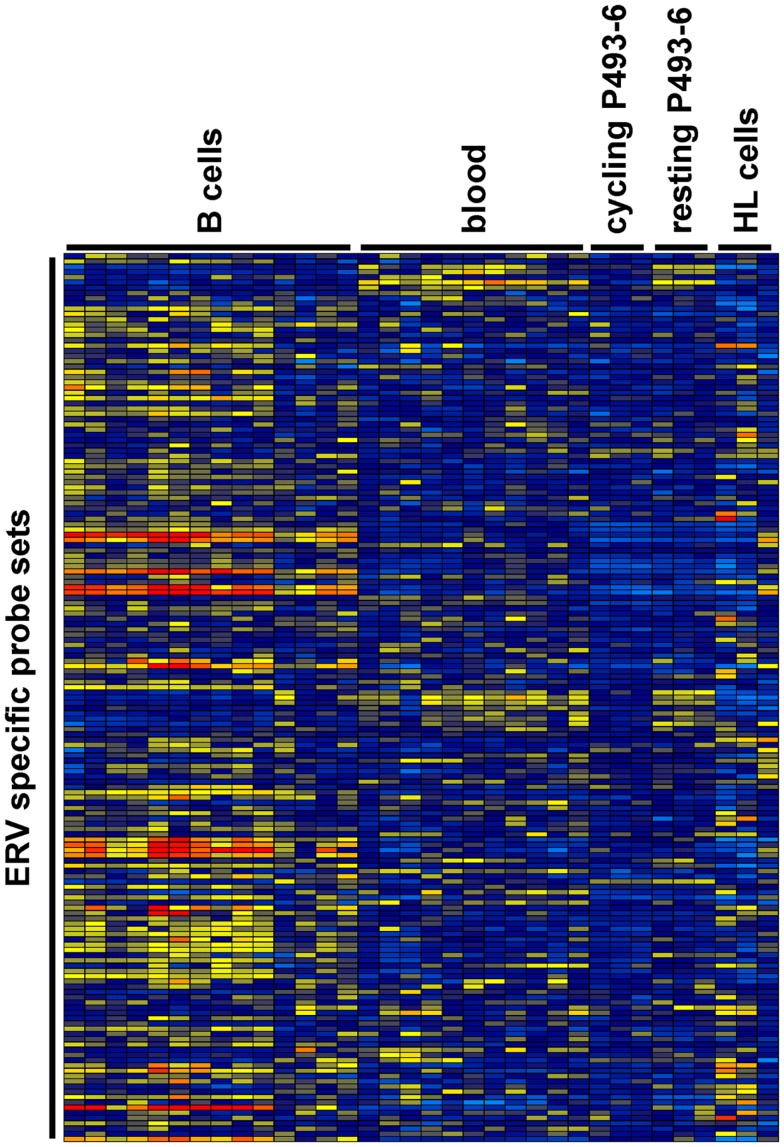
**Heat map of ERV-specific probe sets in the investigated DNA microarray data**. Probe sets with specificity for ERV-related sequences were selected from microarray data from normal blood cells [GEO data set GSE18838 (40)], normal B cells [GEO data sets GSE14352 and GSE5188 (42, 43)], P493-6 cells [GEO data set GSE32219 (41)], and HL cell lines. Signal intensities were visualized using the Genesis software (log2-transformed and mean centered data). The used arrays contain probe sets for the following ERV: *ERV3-1, ERV3-2, ERV9-1, ERV18-1, ERVFC1-1, ERVFH21-1, ERVFRD-1, ERVFRD-2, ERVH-1, ERVH-4, ERVH-6, ERVH48-1, ERVI-1, ERVK3-1, ERVK3-2, ERVK-6, ERVK-7, ERVK-9/-4/-19, ERVK11-1, ERVK13-1, ERVMER34-1, ERVMER61-1, ERVV-1, ERVV-1/-2, ERVW-1*, and*ERVW-6*.

**Figure 2:**
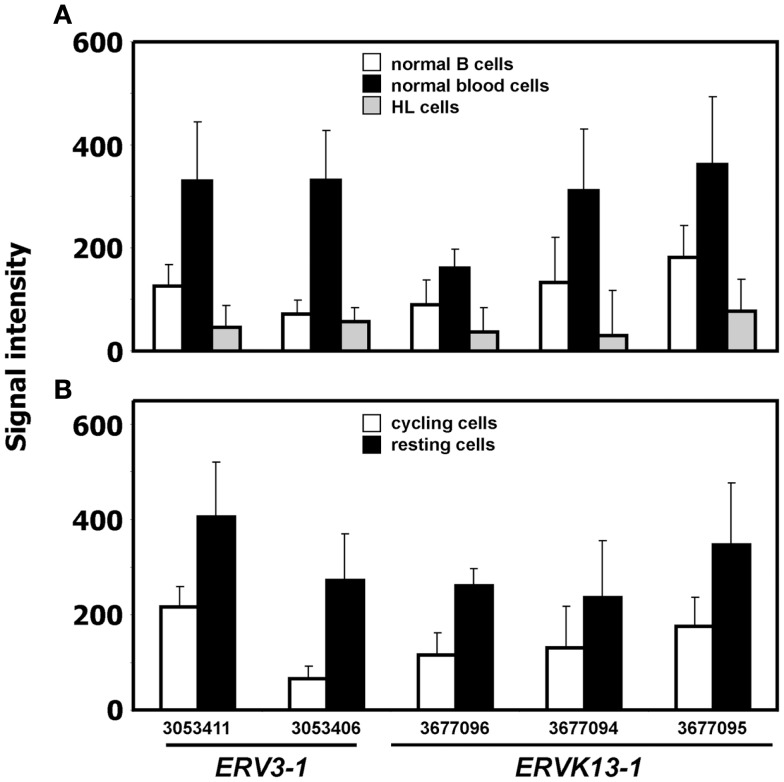
**ERV with significant difference between HL and normal blood samples**. **(A)** Presented are signal intensities (means and standard deviations) of the indicated probe sets (*ERV3-1*: probe sets 303411 and 3053406; *ERVK13-1*: probe sets 3677096, 3677094, and 3677095) in normal blood cells [GEO data set GSE18838 (40)], normal B cells [GEO data sets GSE14352 and GSE5188 (42, 43)], and HL cell lines. **(B)** Signal intensities of the same probe sets in P493-6 cells after treatment with tetracycline (resting cells) or medium without tetracycline [cycling cells; raw data from GEO data set GSE32219 (41)].

We analyzed expression of human ERV in DNA microarray data from HL cell lines HDLM-2, L-428, and L-540 in comparison to normal blood cells, normal B cells, and the conditionally immortalized B cell line P493-6. A total of 169 probe sets with specificity for ERV sequences were analyzed (Figure 1). Signal intensities (RMA normalized, linear values) above 100 were considered to be expressed in the corresponding samples. According to this threshold, HDLM-2 cells expressed 13 different ERV (represented by 31 probe sets), L-428 cells expressed 10 ERV (36 probe sets), and L-540 cells expressed 11 ERV (28 probe sets). In normal blood cells, 13 ERV were expressed (43 probe sets with mean signal intensities above the threshold). Interestingly, the eight ERV that were expressed in all HL cell lines (*ERVFC1-1, ERVH-1, ERVH-4, ERVH48-1, ERVK3-1, ERVK-7, ERVK-9/-4/-19, ERVK13-1*), were also expressed in normal blood cells. In addition, normal blood cells expressed *ERV3-1, ERVK-6, ERVV-1, ERVW-6*, and *ERVMER34-1*. Isolated B cells expressed 15 ERV (45 probe sets with mean signal intensities above the threshold). In addition to all ERV that were found in normal blood, isolated B cells had high signal intensities for *ERV9-1* and *ERVK11-1*. Additional ERV were found only in single HL cell lines: L-428 cells expressed *ERVH-6*; L-540 cells expressed *ERVW-1* and *ERVK3-2*; HDLM-2 cells expressed *ERVFRD-*1 and *ERVFRD-2*. Taken all together, the number of expressed ERV in HL cell lines did not exceed the number of ERV expressed in normal blood or isolated B cells. Probe sets for *ERV3-1* and *ERVK13-1* showed significantly lower (*p* < 0.01) signal intensities in HL cells than in normal blood cells (Figure 2A). We found no ERV that were significantly up-regulated in HL cells. Mean signal intensities for *ERV3-1* and *ERVK13-1* in isolated B cells were lower than the signals in whole blood. It is known from the literature that *ERV3* is up-regulated in cell cycle arrested differentiating cells (45). We tested whether *ERV3* is also up-regulated in cell cycle arrested B cells. For this end, we analyzed DNA microarray data (41) from P493-6 cells that have been treated with tetracycline. Tetracycline switches off expression of *MYC* in this Burkitt lymphoma model cell line leading to cell cycle arrest (34–36). As shown in Figure 2B, we detected up-regulation of all probe sets from Figure 2A in arrested P493-6 cells.

### Validation of *ERV3* expression in cell cycle arrested B cells

**Figure 3:**
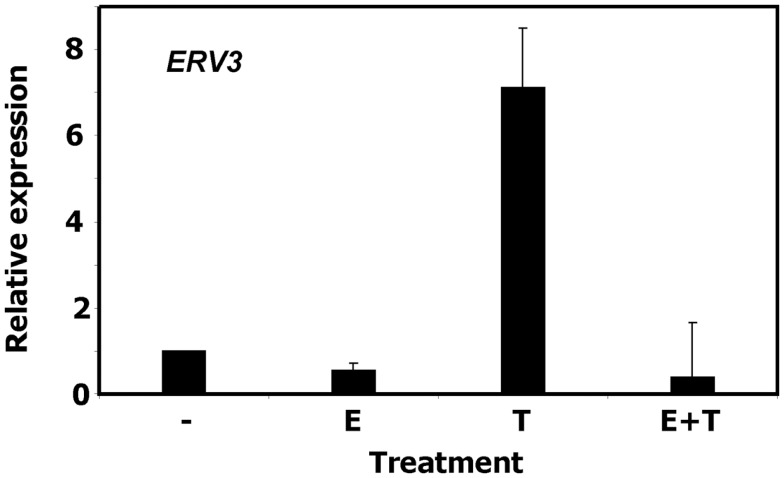
**Expression of *ERV3* in P439-6 cells**. Expression of *ERV3* was analyzed in P439-6 cells under different culture conditions by qRT-PCR. Cells were cultured in the absence or presence of estrogen (E) and/or tetracycline (T). Presented are means and standard errors from duplicate determinations. For comparative analysis, beta actin was used as housekeeping control and the mean of P439-6 cells cultured in medium was set as 1.

To validate the observation of cell cycle dependent regulation of *ERV3* we performed quantitative RT-PCR with P439-6 cells that had been cultured under different conditions. Cells were grown in medium (cycling cells) or after addition of tetracycline (arrested cells). In addition, the same cells were cultured in the presence of estrogen. Estrogen switches on functional EBNA2 in these cells and allows the proliferation in the presence of tetracycline. As shown in Figure 3, incubation of P493-6 cells with tetracycline resulted in marked up-regulation of *ERV3*. Switching on the EBV transformation program by addition of estrogen to tetracycline-treated P493-6 cells inhibited this up-regulation. Similar results were obtained with the conditional EBV-immortalized cell line EREB2-5 (46). This cell line is the parental cell line of P493-6 cells (without exogenous *MYC*) and proliferates only in the presence of estrogen in the culture medium. In this cell line we observed up-regulation of *ERV3* after withdrawal of estrogen (data not shown).

### Regulation of *ERV3* expression in HL cell lines

**Figure 4:**
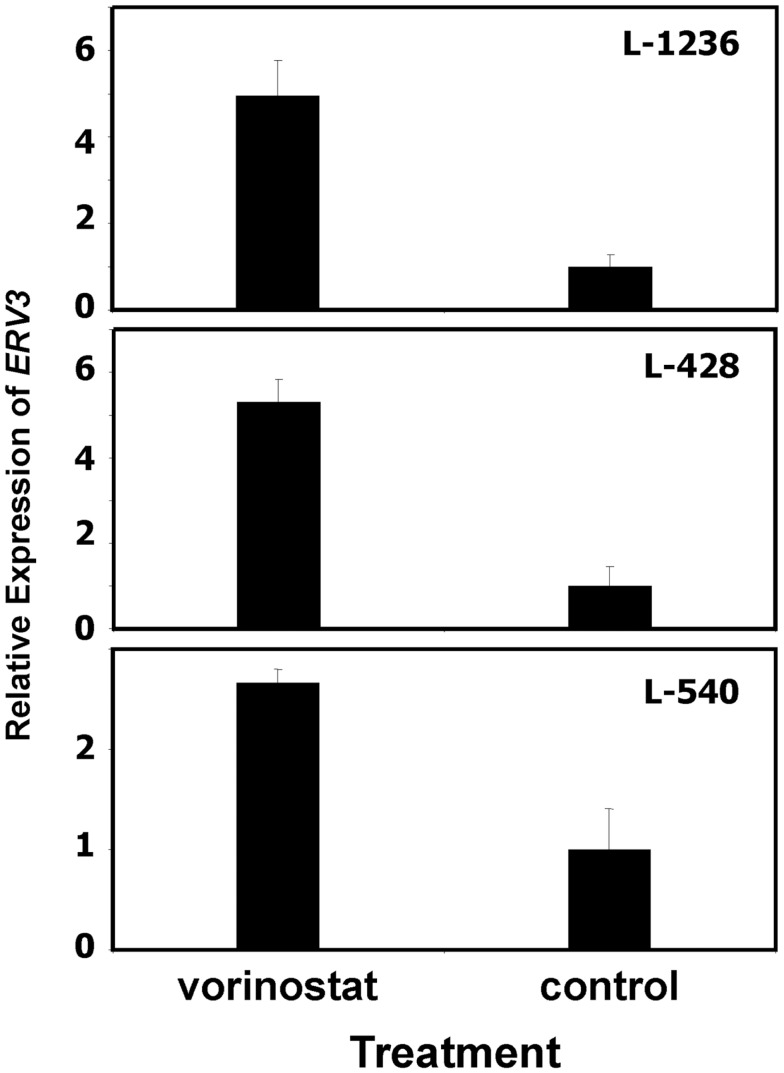
**Incubation of HL cells with the histone deacetylase inhibitor vorinostat leads to increased expression of *ERV3***. Expression of *ERV3* was analyzed in HL cell lines with and without 1 μM vorinostat by quantitative RT-PCR. Presented are means and standard errors from triplicates. Beta actin was used as housekeeping control and the mean of the HL cells without vorinostat was set as 1.

**Figure 5:**
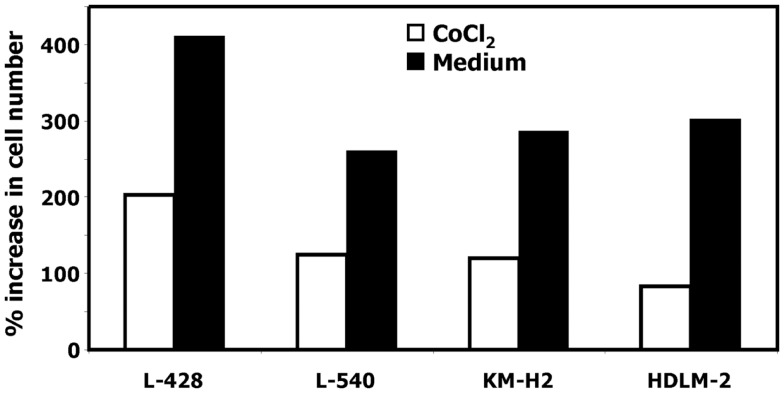
**Incubation of HL cells with cobalt(II) chloride inhibits proliferation**. Cells of the indicated cell lines were cultured in the presence or absence of 200 μM cobalt(II) chloride for 4 days. Thereafter the cell number was determined. Percentage of increase in cell number was calculated as [(final cell number)/(starting cell number) × 100].

**Figure 6:**
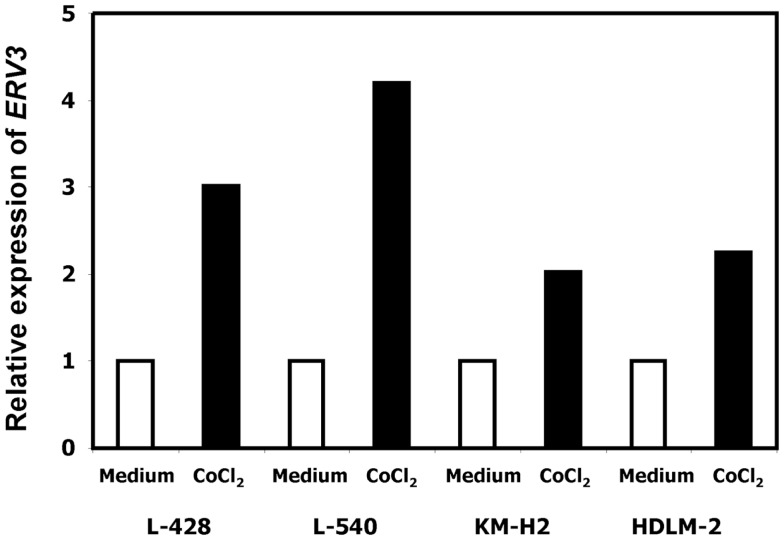
**Incubation of HL cells with cobalt(II) chloride leads to an increased expression of *ERV3***. Expression of *ERV3* was analyzed in HL cell lines L-428, L-540, KM-H2, and HDLM-2 after treatment with 200 μM cobalt(II) chloride or without cobalt(II) chloride by quantitative RT-PCR. Beta actin was used as housekeeping control and the mean of HL cells without cobalt(II) chloride was set as 1.

In our previous work we observed that treatment of HL cells with the histone deacetylase inhibitor vorinostat induces cell cycle arrest (37). Therefore, we asked whether this cell cycle arrest is also accompanied by up-regulation of *ERV3* in HL cells. Figure 4 show the results of this analysis. Incubation of all tested HL cell lines with vorinostat resulted in an increased expression of *ERV3*. We observed that treatment of HL cells with the hypoxia-mimetic CoCl_2_ led to a pronounced inhibition of proliferation (Figure 5). As shown in Figure 6, the expression of *ERV3* again increased when cells were cultured under conditions of inhibited proliferation.

## Discussion

Increased expression of ERV derived sequences has been observed in cancer cells (14, 15, 47–51) and in patients with autoimmune diseases or neurodegenerative diseases (52–54). ERV can act as alternative promoters for adjacent genes (7, 55–58). The resulting fusion transcripts can result in new protein isoforms, or the ERV component of these fusion transcripts can inhibit translation (58). In addition, ERV expression can interfere with the expression of adjacent genes at the level of transcription (59). In cancer cells such interference may lead to the inactivation of tumor suppressor genes. Some ERV-encoded proteins can directly bind and inactivate tumor suppressor genes (60). In our present study we provide preliminary evidence for a differential expression of *ERV3* in HL cells under conditions of growth arrest. The method used for analysis of ERV expression in HL has several limitations. Not all human ERV loci are represented on the arrays and it might be that other ERV are differentially expressed in the investigated cells. In addition, the comparability of our data sets from HL cells and published data sets from normal blood and B cells might also be sub-optimal. However, our results gave no evidence for a general up-regulation of the investigated ERV loci in HL cells. The *ERV3* signals in normal B cells are relatively low. Therefore and based on the limitations of the study, we cannot conclude that the low *ERV3* expression is a specific feature of HL cells. However, up-regulation of *ERV3* in HL cells under conditions of growth arrest suggests that *ERV3* might be an interesting gene for further studies. *ERV3* is unique among ERV as it is considered to be a tumor suppressor (61). *ERV3* is abundantly expressed in the placenta and it is expressed in most other tissues at lower levels (62). Absence of expression in choriocarcinoma was observed (62), and transgenic expression of *ERV3* in choriocarcinoma cells inhibits cell proliferation (63). This growth inhibition is associated with down-regulation of cyclin B and up-regulation of the cyclin dependent kinase inhibitor p21 (63). Expression of *ERV3* is up-regulated during terminal differentiation of leukemia cells and highest in cell cycle arrested cells (45, 64). *ERV3* is a member of an ERV family with more than 40 members, but only *ERV3* has intact ORF for viral proteins (65). The chromosomal location of human *ERV3* is characterized by a high number of pseudogenes (data not shown), and the complete *ERV3* locus is present only in Old World primates with the exception of gorillas (66). Surprisingly, approximately 1% of Caucasians with normal phenotype have mutations in *ERV3* which interrupt the ORF of the *ERV3* envelope (67). This observation suggests that the *ERV3* encoded envelope protein is not critically involved in the physiological function of this gene. Interestingly, read-through transcript between *ERV3* and the neighboring zinc finger protein 117 (*ZNF11*7) have been described (68). Lower expression of these transcripts has been observed in patients with multiple sclerosis (69). The human zinc finger proteins *ZNF107, ZNF138*, and *ZNF92* have high homology with *ZNF117*. Together with other zinc finger proteins these genes form a cluster on human chromosome 7. The physiological function of ZNF117 has not been clarified, but it seems possible that this gene contributes to the biological effects of *ERV3*.

Endogenous retrovirus reactivation might occur only in transcriptionally active regions of the genome. In such cases, ERV reactivation might be only an epiphenomenon of chromatin opening and depends on the presence of adequate competence factors allowing transcription of the ERV. In such a model one would expect that several ERV loci are activated at the same time point. Our results show no evidence for such a general activation of ERV loci in HL. However, our analysis includes only well characterized ERV loci which are detectable by the used microarrays. Reactivation of ERV associated alternative promoter in the *CSF1R* gene seems to be involved in the pathophysiology of HL (7). The identification of such alternative transcription start sites by means of DNA exon microarray analysis requires new bioinformatics tools which are currently being developed in our lab.

The up-regulation of *ERV3* under conditions of cell cycle inhibition and/or terminal differentiation is not specific for HL. Whether such up-regulation occurs only in transformed hematopoietic cells or also in other cell types has to be determined. Up-regulation of *ERV3* in HL cells occurred under conditions which are characterized by increased apoptosis. CoCl_2_ can induce apoptosis in hematopoietic and non-hematopoietic tumor cells (70–73). Similarly, vorinostat and other histone deacetylase inhibitors induce apoptosis in HL cells (74–76). If *ERV3* is a tumor suppressor gene (as suggested by the choriocarcinoma data discussed above), expression of *ERV3* in HL cells and other hematopoietic cells under pro-apoptotic and anti-proliferative conditions might indicate a tumor suppressing activity of *ERV3* also in these cell types. The elucidation of *ERV3* activities in the context of growth inhibition and apoptosis might help the identification of new targets for the treatment of HL and other malignant diseases.

## Conflict of Interest Statement

The authors declare that the research was conducted in the absence of any commercial or financial relationships that could be construed as a potential conflict of interest.
